# Detection of Circulating Serum microRNA/Protein Complexes in ASD Using Functionalized Chips for an Atomic Force Microscope

**DOI:** 10.3390/molecules26195979

**Published:** 2021-10-02

**Authors:** Anna L. Kaysheva, Arina I. Isaeva, Tatyana O. Pleshakova, Ivan D. Shumov, Anastasia A. Valueva, Maria O. Ershova, Irina A. Ivanova, Vadim S. Ziborov, Ivan Y. Iourov, Svetlana G. Vorsanova, Stepan V. Ryabtsev, Alexander I. Archakov, Yuri D. Ivanov

**Affiliations:** 1 Laboratory of Nanobiotechnology, Institute of Biomedical Chemistry, Pogodinskaya St. 10/8, 119121 Moscow, Russia; kaysheva1@gmail.com (A.L.K.); t.pleshakova1@gmail.com (T.O.P.); shum230988@mail.ru (I.D.S.); varuevavarueva@gmail.com (A.A.V.); motya00121997@mail.ru (M.O.E.); i.a.ivanova@bk.ru (I.A.I.); ziborov.vs@yandex.ru (V.S.Z.); alexander.archakov@ibmc.msk.ru (A.I.A.); yurii.ivanov.nata@gmail.com (Y.D.I.); 2 Laboratory of Shock Wave Impacts, Joint Institute for High Temperatures of the Russian Academy of Sciences, Izhorskaya St. 13 Bd.2, 125412 Moscow, Russia; 3 Mental Health Research Center, 117152 Moscow, Russia; ivan.iourov@gmail.com; 4 Veltischev Research and Clinical Institute for Pediatrics, Pirogov Russian National Research Medical University, Ministry of Health of Russian Federation, Taldomskaya St. 2, 125412 Moscow, Russia; svorsanova@mail.ru; 5 Center for Research of Social Systems, 354340 Sochi, Russia; Bob8bob@mail.ru

**Keywords:** microRNA, atomic force microscopy, AFM chip, autism spectrum disorders, time-of-flight mass spectrometry

## Abstract

MicroRNAs, which circulate in blood, are characterized by high diagnostic value; in biomedical research, they can be considered as candidate markers of various diseases. Mature microRNAs of glial cells and neurons can cross the blood–brain barrier and can be detected in the serum of patients with autism spectrum disorders (ASD) as components of macrovesicles, macromolecular protein and low-density lipoprotein particles. In our present study, we have proposed an approach, in which microRNAs in protein complexes can be concentrated on the surface of AFM chips with oligonucleotide molecular probes, specific against the target microRNAs. MicroRNAs, associated with the development of ASD in children, were selected as targets. The chips with immobilized molecular probes were incubated in serum samples of ASD patients and healthy volunteers. By atomic force microscopy (AFM), objects on the AFM chip surface have been revealed after incubation in the serum samples. The height of these objects amounted to 10 nm and 6 nm in the case of samples of ASD patients and healthy volunteers, respectively. MALDI-TOF-MS analysis of protein components on the chip surface allowed us to identify several cell proteins. These proteins are involved in the binding of nucleic acids (GBG10, RT24, RALYL), in the organization of proteasomes and nucleosomes (PSA4, NP1L4), and participate in the functioning of the channel of active potassium transport (KCNE5, KCNV2).

## 1. Introduction

Autism Spectrum Disorders (ASD) pertain to complex disintegrative disorders of mental development. They are characterized by impaired social interaction, communication, stereotypical behaviour and social maladjustment. Worldwide, about 1% of people suffer from ASD, which are five times more common in men than in women [1,2]. Genetic modifications, metabolic disorders, environmental factors, and their combination are considered to be potential causes of ASD. As is known, medical and pedagogical interventions are effective in the early stages of development of the disease—that is, in the first years of a child’s life. However, due to the lack of specific serological markers of autism, its early diagnosis at the preverbal stage of child development is difficult [3]. Currently, ASD in children are only diagnosed based on behavioural symptoms. A better understanding of the causes of autism can be achieved by identifying molecular biomarkers (proteins, metabolites, circulating noncoding RNAs) of the disease along with behavioural responses. Such an approach is believed to provide early diagnosis of ASD [3].

Since the first description of ASD cases in the early 1940s, a considerable amount biomedical research, aimed at the identification of serological markers of autism, was performed. It is notable that in recent years, the identification of protein markers of ASD has attracted growing attention. Previously, in our pilot study, we demonstrated the efficient application of ultra-high resolution mass spectrometry for the comparative protein profiling of nine depleted serum samples, five of which were obtained from children with ASD [4]. We showed that the protein composition of serum samples from children with autism differed from that of control samples. In addition, we identified a small group of 13 proteins common to all samples of children with ASD. This group of proteins included attractin, protein 1 containing the LIM domain, protein S100-A6, and others. Researchers are extremely interested in identifying serological markers of ASD, and in the past decade, over 200 proteomic studies involving biological samples of children with ASD have been published. The most popular candidate markers of ASD include proteins, peptides, auto-antibodies, cytokines, and oxidative stress proteins, which are likely to be associated with the development of ASD in children [2].

Analysis of literature sources, performed in order to identify probable candidate markers in the pool of circulating microRNA in blood samples of patients with ASD, made it possible to form a list of noncoding microRNAs, associated with the development of the disease. This list includes miR-106, miR-140, miR-320a, miR-19b, miR-146a, miR-494, miR-664-3p and others [5]. The analysis of the literature indicated the identified microRNA in this list to be involved in the regulation of gene expression, reprogramming of cell metabolism, regulation of proliferation processes, cell differentiation, etc. MicroRNA expression signatures are altered in the brain, blood, saliva, and olfactory progenitor cells of patients with ASD. The ability of miRNAs to regulate a wide range of molecular signaling pathways in response to an environmental stimulus (epigenetic factor) plays an important role in the development of ASD. Besides, the availability and extracellular stability of microRNAs make them ideal candidates for the search for new biomarkers. Moreover, a population of microRNAs circulates in the blood and can be found in protein complexes [6]. The high stability of circulating miRNAs under extracellular conditions (at high level of hydrolase enzymes) is explained by the formation of macromolecular complexes, including extracellular vesicles (EV), Argonaut 2 proteins (AGO2), or high-density lipoproteins (LPHD) [7]. However, the origin and biological significance of the forms of miRNA-containing extracellular complexes are still poorly understood. 

To date, biomedical research lacks a “gold standard” method for miRNA detection in biological samples [8]. For the detection of microRNAs in complex biological samples, various analytical platforms are used, depending on the scientific research task. Electrochemical, electrical (including potentiometric) [9,10,11,12,13,14,15,16] and surface plasmon resonance (SPR)-based methods [16] were reported. Next generation sequencing (NGS) [17], microarray profiling [18] and real-time reverse transcription PCR (RT-qPCR) [8,17] are employed most often. Each of these technologies has its own advantages and disadvantages [12]. In a number of scientific studies on the detection of microRNAs in cancer cell lines, and in human biological samples, a low level of correlation between the results obtained by NGS, microarray profiling, and RT-qPCR was demonstrated [18,19,20,21,22,23].

It should be noted that technologies for direct microRNA detection are characterized by low sensitivity and require a high concentration of microRNA in the sample under study. On the other hand, the disadvantage of using amplification-based technologies consists in reading errors in case of short microRNA sequences [24]. The microarray-based profiling is characterized by low specificity and concentration sensitivity (one analysis requires 0.2 to 2 μg of total microRNA), and cross-hybridization of microRNA is also possible [8]. On the contrary, qRT-PCR is characterized by high sensitivity and specificity, as well as by the wide dynamic range of detectable concentrations (of about seven orders of magnitude), However, since miRNA sequences are short (about 22 to 24 nucleotides), applicability of this approach is limited [25,26]. Namely, in the case of short microRNAs, the efficiency of PCR is reduced due to the low melting point, and this leads to errors, especially upon the analysis of biological fluids [27,28]. Due to this fact, in several studies, additional sample preparation procedures (ligation step with an extension sequence) were used in order to lengthen the target molecules upon analysis of biological fluids [27,29]. In addition, PCR-based methods are sensitive to sample contamination, since amplification is employed [30]. NGS technology is characterized by high specificity. The analysis, however, requires a relatively large amount of microRNA (at least 10 μg of total RNA). NGS is also characterized by a high risk of obtaining false negative results in the case of the detection of low-abundance microRNAs in biological samples [8]. In addition, NGS technology is still quite expensive.

In this regard, the development of novel technological approaches for the detection of microRNAs in biological samples represents an urgent task of biomedical research. In this regard, the use of molecular detectors, which, in theory, have no restrictions on concentration sensitivity, and allow one to detect target molecules at low concentrations, is promising [31]. Molecular detectors do not require amplification of the signal from target molecules, and in the case of an atomic force microscope, allow one to visualize and count single biological molecules on the surface of functionalized AFM substrates (AFM chips) [31,32]. Such detectors are free from the above-mentioned disadvantages, which are typical for the commonly used microRNA detection systems.

Herein, a novel experimental approach, which combines atomic force microscopy (AFM) with mass spectrometry (MS), is proposed. This approach combines the advantages of the two platforms. On the one hand, the AFM-based molecular detector allows one to visualize and count single macromolecular complexes on the surface of functionalized substrates for AFM (AFM chips), where the target biological molecules are efficiently concentrated. In this approach, neither the amplification of the signal from the target molecules, nor the use of additional labels (fluorescent or enzymatic) is required. On the other hand, MS-based methods allow one to identify objects visualized by AFM [32]. In addition, ultra-high-resolution AFM scanning allows one to monitor the efficiency of procedures (including the quality of the chip surface, the efficiency of immobilization of molecular probes, and the formation of affinity complexes) throughout the experiment. Thus, the number of false negative results is considerably reduced, making it possible to quickly select optimal experimental conditions for the detection of various biological macromolecules. In comparison with other methods employed for microRNA detection, the combined label-free AFM-MS approach, proposed herein, allows one to obtain information not only about the target molecules, but also about partner proteins, which are probably associated with the target molecules under study in biological samples. Thus, the combined AFM-MS approach allows one to expand the range of solved experimental biomedical problems.

In the present research, molecular fishing of target miRNAs as a part of macromolecular protein complexes, has been performed with the use of AFM chips, functionalized with oligonucleotide probes. The chip surface was sensitized with immobilized oligonucleotide molecular probes, specific against the target miRNAs. The so-sensitized AFM chips were incubated in the analyzed serum samples. After the incubation, AFM visualization and detection of probe/target molecular complexes, formed on the surface of the AFM chip, and mass spectrometric identification of the captured protein components were performed.

In this study, with the example of chips for an atomic force microscope, we draw the readers’ attention to expanding the possibilities of using functionalized surfaces for the detection of macromolecular complexes, which are often formed in complex samples of biological origin. With the example of the detection of target microRNAs, it is possible to analyze possible nucleoprotein complexes that are formed in vivo, including a protective natural mechanism for maintaining the integrity of small nucleic acid sequences, which have an important biological function.

## 2. Materials and Methods

### 2.1. Chemicals

Dulbecco’s modified phosphate buffered saline (PBSD; 10 mM, pH 7.4), acetonitrile and 3,3′-dithiobis (sulfosuccinimidyl propionate) (DTSSP) cross-linker were purchased from Thermo Fischer Scientific (Waltham, MA, USA). (3-aminopropyl) triethoxysilane (APTES), ammonium bicarbonate and dimethyl sulfoxide (DMSO), trifluoroacetic acid (TFA) and α-cyano-4-hydroxycinnamic acid (HCCA) were purchased from Sigma-Aldrich (St. Louis, MO, USA). Isopropanol and formic acid were purchased from Acros Organics (Geel, Belgium). Deionized water from the “Simplicity UV” system (Millipore, Inc, Molsheim, France) [32]. Porcine trypsin was purchased from Promega Corporation (Madison, WI, USA). 

Oligonucleotides, whose sequences were complementary to the target microRNAs, were used as molecular probes immobilized on the surface of the sensor areas of the AFM chips. All these oligonucleotide probes had an additional NH_2_-(T)_10_-linker on their 5′-end in order to provide their covalent immobilization onto the amino mica surface of the AFM chips. The oligonucleotide molecular probes were purchased from Evrogen (Moscow, Russia). The nucleotide sequences of the molecular probes, complementary to the ASD-associated target microRNAs, are listed in Table 1.

In addition, oligonucleotides, which were known to have no specificity against the target microRNAs, were used in the experiments as control molecular probes. These control probes were complementary to miRNAs associated with a different pathology (ovarian cancer). The nucleotide sequences of these control molecular probes are listed in Appendix A Table A1.

### 2.2. Study Participants

Seven children with ASD (9 ± 2 years old) and four healthy parents (31.5 ± 18 years old) were initially selected for this study. The control group of apparently healthy volunteers represents mothers of children with ASD and a healthy son of a family (Table 2). Children in the ASD group were recruited from State educational institution of Krasnodar region «The Boarding School No. 2», Russia. The inclusion criteria consisted in a diagnosis of ASD based on the ICD-10 (International Classification of Diseases, Tenth Revision)/DSM-5 (Diagnostic and Statistical Manual of Mental Disorders, Fives Revision) criteria determined by a child psychiatrist. The ASD diagnosis was further confirmed by the following questionnaires: Autism Diagnostic Interview (ADI-R), Autism Diagnostic Parents Checklist (ADPC) [33].

The patients with severe somatic and neurological pathologies, as well as patients taking long-term pharmacotherapy with an established ASD diagnosis were excluded from the study. At the time of our research, patients with ASD were treated with behavioural therapy, designed to improve communication and relationships with others. In our study, we analysed samples from children with the following diagnosis (DSM-10): atypical autism (F84.1) [33].

### 2.3. Preparation of Serum Samples

We studied four control serum samples from healthy volunteers and 13 samples from children with ASD. Serum samples were prepared from venous blood, taken from either male ASD patients or healthy male volunteers on an empty stomach after an overnight fast. The samples were provided by the Mental Health Research Center and Laboratory of Molecular Cytogenetics of Neuropsychiatric Diseases, Veltischev Clinical Pediatric Research Institute, Pirogov Russian National Research Medical University (Moscow, Russia). Written informed consents were obtained from the individuals, who provided the samples, prior to their participation in the study [33].

The blood was collected into pre-chilled tubes containing ethylenediaminetetraacetic acid (EDTA), mixed rapidly, and centrifuged at 4 °C at 1500 revolutions per minute (rpm) for 10 min; and the serum samples were immediately collected and frozen. After centrifugation, the supernatant in the tubes was carefully collected with an automated pipette and placed into 2 mL cryovials [32]. The samples were stored at −80 °C and were not subjected to repeated freeze/thaw cycles.

For the AFM-based analysis, the serum samples were diluted with the addition of 10 µL of serum to 990 µL of PBSD buffer with subsequent 15-min incubation in a shaker at 600 rpm in order to achieve even distribution of sample components over the entire volume of the solution [33].

### 2.4. Preparation of AFM Chips

AFM chips were fabricated from rectangle 7 mm × 15 mm pieces of muscovite mica (SPI, USA), treated according to techniques described elsewhere [33,34,35]. Briefly, the treatment of the mica comprised the following steps: silanization; formation of an array of metallic marks onto the mica surface; formation of an array of sensor areas with immobilized molecular probes against the target miRNAs.

All AFM chips used in the experiments were subjected to preliminary quality control procedures in order to ensure proper roughness of the chip surface. The quality control was performed by AFM on the following steps:

- before the immobilization of oligonucleotides (the height of objects should not exceed 0.5 nm);
- after the immobilization of oligonucleotides (the height of the objects should not exceed 1.5 nm);
- after the incubation of the AFM chip in the analyzed samples and washing procedures (the amount of AFM-visualized objects with heights greater than 1.5 nm should be at least 1000 per 400 μm^2^).

The AFM chips were silanized with APTES in vapor phase as described elsewhere. Subsequent formation of metallic marks on the silanized AFM chip surface was carried out by DC magnetron sputtering deposition of chromium and tungsten layers in argon plasma following the technique by Shumov et al. [35] with an Orion-3 magnetron sputtering system (AJA International, Inc., North Scituate, MA, USA).

**Figure 1:**
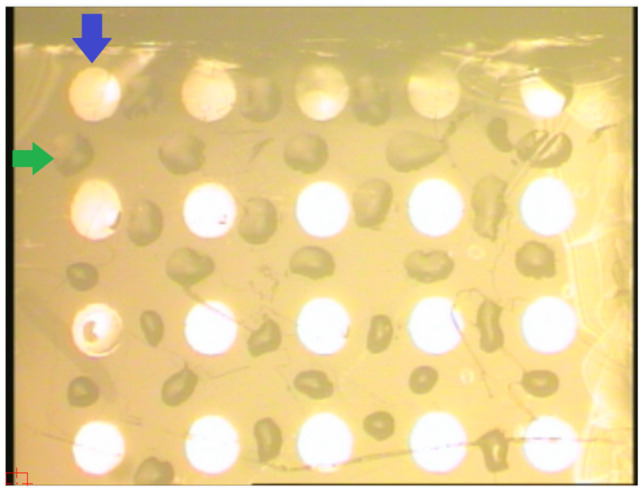
Optical image of the AFM chip surface bearing an array of sensor areas. Blue arrow indicates the magnetron-sputtered metallic mark. Green arrow indicates the micro-drop of the solution of an oligonucleotide probe dispensed with a Piezorray low-volume high-accuracy dispensing system (Perkin Elmer, Inc., Waltham, MA, USA). Each type of oligonucleotide probes was dispensed onto the surface of three separate sensor areas between the metallic marks. After the incubation of the AFM chip in the analyzed sample and subsequent washing, the AFM probe is positioned above the selected sensor area for scanning using the built-in AFM’s video system, and with the aid of a photographic image of the chip with the dispensed micro-drops.

The molecular probes against the target microRNAs were immobilized onto the AFM chip surface, preliminarily activated with DTSSP cross-linker, in the form of an array of sensor areas analogously to the previously described technique [36]. Briefly, immediately after the surface activation, solutions of the oligonucleotide probes were precisely dispensed onto the chip surface in the form of an array of 3-nL micro-drops employing a Piezorray non-contact low-volume high-accuracy dispensing system (Perkin Elmer, Inc., Waltham, MA, USA). The solutions for the dispensing were prepared by mixing equal volumes of aqueous solutions of the oligonucleotide probes (20 µM), PEG (10% *w*/*v*) and glycerol (10% *v*/*v*). The micro-drops were incubated on the chip surface for 30 min in a humid chamber, and then extensively washed with ultrapure water in a shaker at 37 °C and 650 rpm for 45 min. In this way, an array of sensor areas was formed on the AFM chip surface, so that different oligonucleotide types were immobilized in each area. Each oligonucleotide type was immobilized in three separate sensor areas of the array, as specified in Table 1. Aside from the areas with immobilized oligonucleotides, control areas without immobilized oligonucleotides were also present on the chip surface. Figure 1 displays an optical image of the array of sensor areas on the AFM chip.

Molecular fishing of target microRNAs from the serum samples to be analyzed was performed in the following way. The AFM chip, bearing an array of sensor areas with immobilized oligonucleotide molecular probes, was incubated in 1 mL of diluted serum for 60 min in a Thermomixer Comfort shaker (Eppendorf, Hamburg, Germany) at 37 °C and 600 rpm. After the incubation, the chip was washed once in 1 mL of washing solution (0.01% aqueous solution of Emulgen 913), and then twice in 1 mL of ultrapure water. Each washing was performed in a shaker at 37 °C and 600 rpm for 30 min. After washing, the chip was air-dried and then subjected to AFM measurements [33].

### 2.5. AFM Measurements

AFM scanning was performed in tapping mode in air with a Titanium atomic force microscope (NT-MDT, Zelenograd, Russia; the microscope pertains to the equipment of “Human Proteome” Core Facility of the Institute of Biomedical Chemistry, supported by Ministry of Education and Science of Russian Federation, agreement 14.621.21.0017, unique project ID: RFMEFI62117X0017) equipped with NSG03 silicon cantilevers with gold reflecting coating (TipsNano, Zelenograd, Russia; typical resonance frequency 47 to 150 kHz, tip curvature radius 10 nm) [37].

For each scanned sensor area, at least five 5 µm × 5 µm frames with 256 × 256 resolution were obtained. The AFM operation, obtaining AFM images, image processing (flattening correction etc.) and the data export into ASCII format was carried out using standard NOVA Px software (NT-MDT, Zelenograd, Russia) supplied with the atomic force microscope as described elsewhere [33]. The heights of objects in the AFM images were measured using a specialized software for AFM data processing, developed in the Institute of Biomedical Chemistry (Rospatent registration No. 2010613458). The cut-off level (noise level) amounted to 500 objects per 400 μm^2^ area [32]. The height of the AFM-imaged objects was used as the criterion for the determination of their dimensions.

### 2.6. Preparation of AFM Chips for Mass Spectrometric Analysis

Earlier, in our studies concerning the detection of proteins at low (10^−9^ M and lower) concentrations in purified solutions and in samples of biological fluids (serum), we selected optimal conditions for hydrolytic cleavage of proteins on functionalized surfaces of AFM chips. Trypsinolysis of protein objects was performed directly on the surface of AFM chips, whereon 10 μL of an incubation solution (comprising 0.1 μM of modified trypsin in 100 mM bicarbonate buffer (pH 7.4) was applied [38].

### 2.7. MALDI-TOF Measurements

Protein identification was carried out using Autoflex III TOF mass-spectrometer (Bruker Daltonik GmbH, Bremen, Germany), equipped with a nitrogen laser with an emission wavelength of 337 nm.

Mass spectrometric measurements were performed according to the technique reported in our previous papers [38,39].

### 2.8. Schematic of the AFM-MS Analysis

**Figure 2:**
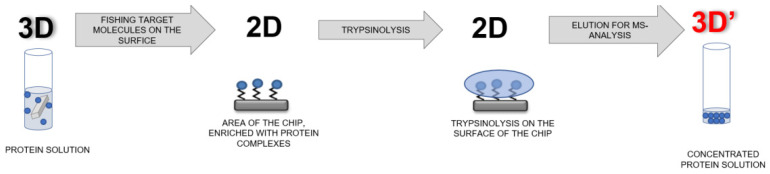
Schematic representation of mass spectrometric analysis of protein complexes concentrated on the surface of AFM chips.

AFM-MS analysis of objects captured onto the sensor areas of the AFM chips during the incubation in the analyzed samples was performed according to the following scheme (shown in Figure 2).

At the first stage of the analysis, the surface for the AFM chip is sensitized—namely, the covalent immobilization of oligonucleotide molecular probes on the surface of sensor areas of the chip is performed. Surface sensitization is described in Section 2.4. Then, the sensitized chip is incubated in the analyzed sample and, after subsequent washing procedures, is transferred to the mass spectrometry section in order to carry out preliminary chip preparation procedures for mass spectrometric measurements. Such procedures include trypsinolysis on the chip surface, followed by elution with a small volume (10 μL) as described in Section 2.6. Then, mass spectrometric measurements are performed.

## 3. Results

In the experimental part of our study, we used AFM chips, fabricated on the basis of silanized mica substrates with terminal primary amine groups exposed onto their surface. On the surface of these substrates, an array of sensor areas with covalently immobilized oligonucleotide molecular probes against target ASD-associated microRNAs was formed. Molecular AFM-based fishing was carried out by biospecific capturing of the target microRNAs onto the surface of the sensor areas in the course of incubation of the chips in the analyzed serum samples. Such an approach allowed us to concentrate the target molecules from the volume of the analyzed samples in a small sensor area of the chip.

Totally, 11 blood serum samples were analyzed in the study, including 7 blood samples from children with ASD (F84.1) and 4 samples obtained from apparently healthy mothers of children. On each step of the experiment (namely, silanization of the AFM substrates; immobilization of the molecular probes; molecular fishing of the target microRNAs on the AFM chip surface), AFM visualization was carried out in order to control the surface quality of: (a) mica substrates, which should be smooth, and the height of the visualized objects should not exceed 0.5 nm; (b) the chip surface after the immobilization of oligonucleotides, the height of functionalized objects surfaces should not exceed 1.5 nm; and (c) the chip surface after its incubation in the analyzed samples and washing procedures, at least 1000 objects with a height exceeding 1.5 nm should be visualized on a 400-μm^2^ area of the chip.

**Figure 3:**
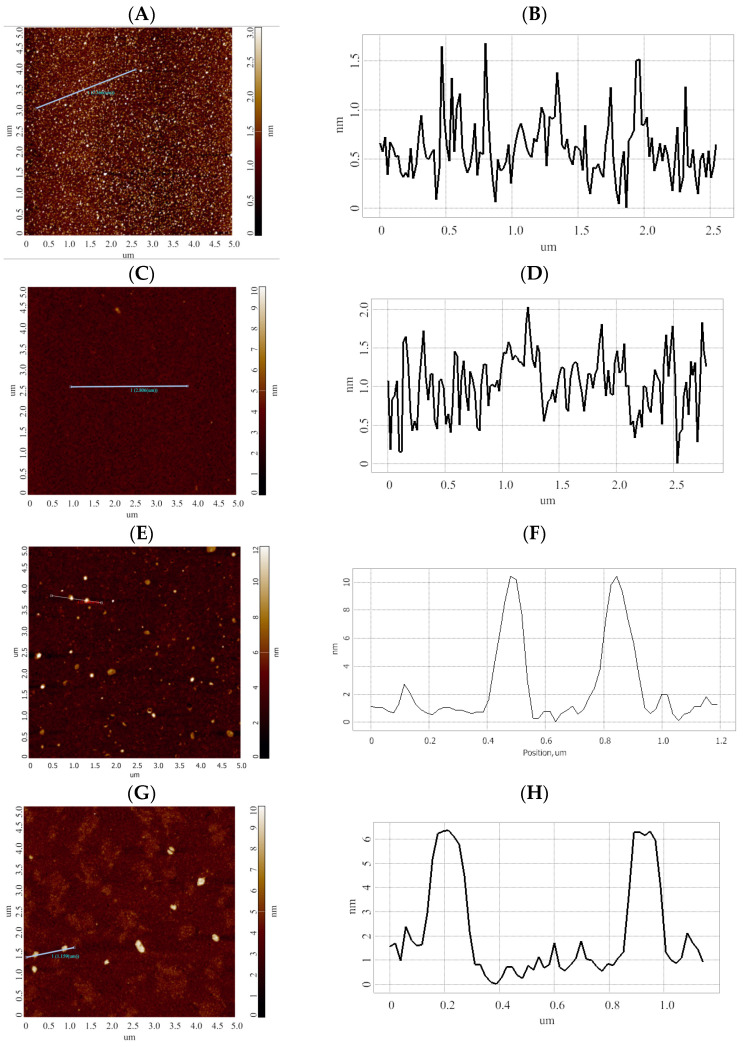
Typical AFM images (left) and cross-section profiles (right) obtained in the sensor areas AFM chip surface after the immobilization of oligonucleotide probes (**A**,**B**); of the sensor area sensitized with molecular probes specific against microRNA associated with ovarian cancer (non-specific probe, negative control) after the incubation in the sample of ASD patient (**C**,**D**); after the incubation in the serum of an ASD patient sample #7 (**E**,**F**); after the incubation in the serum sample #8 of a conditionally healthy child (**G**,**H**). Left panels display the AFM images, while right panels display cross-section profiles, corresponding to the lines shown in the respective AFM images.

As a result of the incubation of AFM chips in serum samples of the two comparison groups, differences in the number and average heights of objects formed on the surface of the sensor areas of the AFM chips were observed. Namely, after the incubation of the AFM chips in the samples of children with ASD, objects with an average height of 10 nm were formed. At the same time, after the incubation in the samples of healthy participants, the height of visualized objects amounted to only ~6 nm. Figure 3 displays typical AFM images (left) and corresponding cross-section profiles (right) obtained on the following steps: after the immobilization of oligonucleotide probes (Figure 3A,B); in the areas sensitized with molecular probes against microRNA associated with ovarian cancer (non-specific probe, negative control) after the incubation of the AFM chip in the sample of an ASD patient (Figure 3C,D); after the incubation of the AFM chip in the serum sample #7 of an ASD patient (Figure 3E,F); after the incubation of the AFM chip in the serum sample #8 of a conditionally healthy child (Figure 3G,H).

As one can see from Figure 3, both after the immobilization of oligonucleotide molecular probes (Figure 3A) and in the case of negative control with the non-specific molecular probe (Figure 3C), the height of objects visualized on the chip surface does not exceed 1.5 nm. Based on our previous studies, we assume that the size of the visualized microRNAs is comparable to that of oligonucleotides with similar molecular weight and nucleotide sequence length, and makes up ~0.9 nm [40]. At the same time, the height of protein/oligonucleotide complexes is 1.6 ± 0.1 nm [40]. The height of objects corresponding to the gp120 (*Mw* = 120 kDa)/antibody (*Mw* = 150 kDa) protein-protein complex is known to be 1.8 to 2.5 nm [40]. In the literature, it is also reported that, upon AFM scanning in air, the heights of AFM images of supramolecular structures of RNA molecules (‘kissing-loop’ RNA and tectoRNA) adsorbed onto mica have heights of 1.2 ± 0.2 nm and 0.9 ± 0.1 nm, respectively [41]. In [42], the heights of AFM images of double-stranded and triple-stranded DNA were reported to be 0.4 ± 0.1 nm and 0.7 ± 0.2 nm, respectively. We assume that the heights of objects (6 nm and 10 nm), which are much higher than expected, most likely correspond to nucleoprotein complexes formed on the chip surface. It should be noted that herein, we did not solve the problem of mass spectrometric detection of target microRNAs for the following reasons. MALDI-MS detection of nucleic acids in biological samples (serum) is difficult due to the formation of numerous types of adducts with cations, which are present in the medium at high concentrations. The formation of adducts critically lowers the resolution of the MALDI-MS method and requires additional desalination procedures—for instance, with the use of C18 Zip-Tips [43,44,45,46]. At the same time, in a number of previously published papers, we showed that the approach of bio-specific fishing of proteins onto a functionalized AFM chip surface makes it possible to efficiently concentrate proteins in an amount sufficient for subsequent mass spectrometric analysis [39,40,47]. In this connection, we have performed proteomic MALDI-MS analysis of protein components, concentrated on the functionalized surfaces of AFM chips after the incubation in the analyzed serum samples.

The analysis of protein components in the composition of AFM-visualized objects was carried out using a MALDI-TOF mass spectrometer. On the surface of sensor areas of AFM chips, at least 10 cellular proteins were revealed after the incubation in samples of individuals from both comparison groups (Figure 1 and Figure 2). The proteins are characterized by functional activities, which probably determine the formation of macromolecular protein-nucleotide complexes: namely, these proteins are involved in the binding of nucleic acids (GBG10, RT24, RALYL), the organization of proteasomes and nucleosomes (PSA4, NP1L4), and the functioning of active potassium transport channel (KCNE5, KCNV2). In terms of their physicochemical properties, proteins differ in molecular weight (from 7 to 62 kDa), isoelectric point (from 4.6 to 9.5), and aliphatic index (from 65 to 100%). As expected, proteins with pI < 7.4 (physiological serum pH) are membrane proteins (KCNE5 and KCNV2) or circulate in the bloodstream as components of vesicles (PSA4, NP1L4). The aliphatic index reflects the relative content of hydrophobic amino acid residues and the volume occupied by the amino acid side groups. Proteins with a high aliphatic index (70 or greater) are thermally stable.

Figure 3 displays a histogram indicating the frequency of the occurrence of proteins, revealed in the analyzed samples by MS analysis.

**Figure 4:**
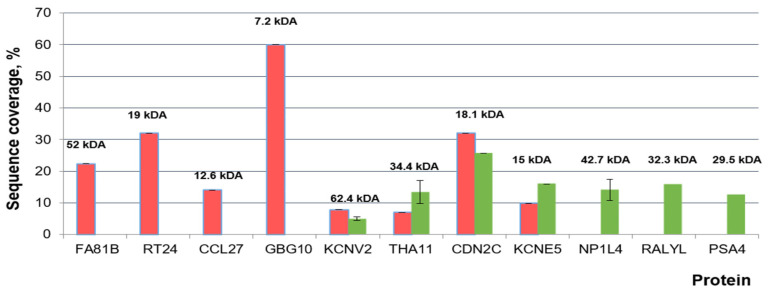
The bar graph displays the sequence coverage of proteins in the MALDI-TOF MS analysis. Proteins found on the chip surface, functionalized with oligonucleotides after incubation in blood samples from ASD patients (red bars, *n* = 7) and healthy volunteers (green bars, *n* = 4). Indicated are the values of the standard deviation of the measurement results, the molecular weight of the proteins.

The bar graph presented in Figure 4 indicates that proteins characteristic for the samples of children with ASD (GBG10, FA81B, RT24, CCL27) were revealed on the surface of the chips, functionalized with probes against ASD associated miRNAs.

At the same time, proteins characteristic for the samples of healthy volunteers (PSA4, NP1L4, RALYL) were revealed with sequence coverage shown in Figure 4. CDN2C, KCNV2, THA11 and KCNE5 were revealed in both comparison groups (Table 3). The coverage of the identified peptides and adducts—that is, the peptide fragments modified with potassium (K^+^) and sodium (Na^+^) cations-ranged from 10 to 60%. Taking into account possible modifications of peptide fragments with Na^+^ and K^+^ increases the reliability of the mass spectrometric identification [48].

**Figure 5:**
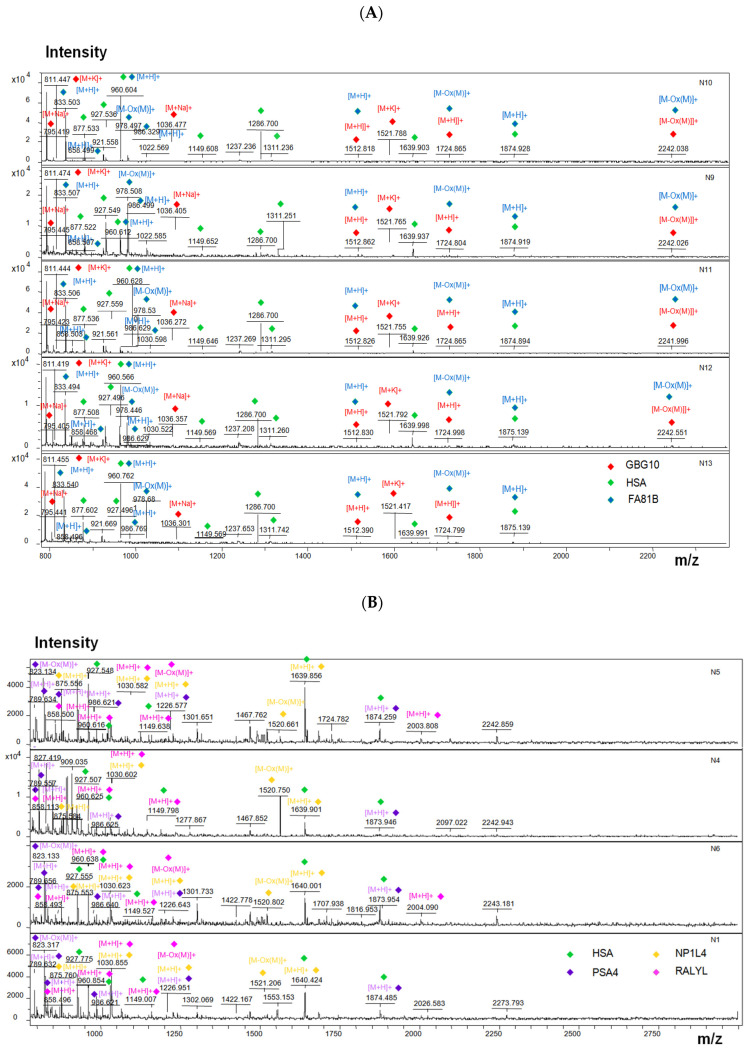
Typical MALDI-TOF spectra for chips with a surface functionalized with oligonucleotides after incubation in serum samples from children with ASD (**A**), or in serum of apparently healthy study participants (**B**). Coloured markers indicate revealed proteins: [M + H] + indicates intact protein peptide, [M + K] +/[M + Na] + indicates intact peptide adduct, [M-Ox (M)] + indicates intact peptide taking into account a post-translational modification–methionine oxidation.

Figure 5 displays typical MALDI-TOF spectra obtained upon the MS analysis of objects, captured on the surface of AFM chips after their incubation in the samples of individuals from both comparison groups. Namely, Figure 5A displays typical MS spectra obtained after the incubation of the chips in samples of children with ASD, while Figure 5B illustrates the case the samples of conditionally healthy volunteers. A high similarity between the composition of the m/z characteristics of the samples within each comparison group (intragroup similarity) was observed, while the composition of the m/z characteristics obtained for the two comparison groups was different.

Serum albumin tryptic peptides were identified in both groups of samples (green marker in Figure 5). Peptides of guanine nucleotide-binding protein and FAM81B protein were revealed on the surface of AFM chips after their incubation in the samples of ASD patients. Proteasome subunit alpha type-4 (PSA4), nucleosome assembly protein 1-like 4 (NP1L4), RNA-binding Raly-like protein (RALYL) were revealed after the incubation of the chips in the samples of healthy volunteers.

## 4. Discussion

In the present study, an approach for the revelation of the macromolecular protein-nucleotide complexes, specifically or non-specifically adsorbed onto the surface of mica AFM chips, has been proposed. In this respect, the AFM chip is considered as an affinity reagent, whose surface is sensitized with oligonucleotide molecular probes, specific against microRNAs associated with the development of ASD. In the course of incubation of these chips in serum samples, target microRNA molecules and protein–nucleic acid macrocomplexes are concentrated on the AFM chip surface. Protein components of macromolecular complexes are subsequently revealed by MALDI-TOF-MS.

Herein, we have used seven types of oligonucleotide molecular probes, whose sequence was complementary to that of the target six microRNAs: miR-106a, miR-19b-3p, miR-494, miR-15b-5p, miR-320a, and miR-195-5p. In the literature, these microRNA types were shown to be associated with the development of ASD in children [49].

MicroRNAs represent small (only 17 to 25 nt) non-coding RNAs, which are involved in the regulation of translation of the coding messenger RNAs (mRNAs). Mature microRNAs are included in RISC (RNA-Induced Silencing Complex), which contains Dicer and Argonaute proteins (AGO), for subsequent degradation of the target mRNA or suppression/activation of mRNA translation. To date, it is known that microRNA can cross the blood-brain barrier, and can be detected in the serum of patients with developmental disorders of the nervous system and neurological disorders as part of microvesicles released by neurons and glial cells, macromolecular protein and lipoproteomic complexes [50].

Previously, in numerous studies, it was shown that in ASD patients, the signatures of miRNA expression are changed in brain, as well as in serum and in other biological fluids [38]. MicroRNAs, studied in this work, are associated with the development of ASD. According to the literature data, miR-106a [5,49,51], miR-19b-3p [52,53,54,55], miR-494 [52,53,54], miR-15b-5p [5,55], miR-320a [5], and miR-195-5p [55] represent the most overlapping dysregulated differentially expressed non-coding RNAs with significant diagnostic values.

In this way, Abu-Elneel et al., (2008) showed that miR-106a is differentially expressed in the cerebellar cortex of patients with ASD, as compared with conventionally healthy study participants [5]. A change in the content of this non-coding RNA in serum was also observed in patients with astrocytoma as was shown by Zhi et al. [51]. Similarly, an increase in miR-19b levels in samples of serum and brain tissue (Brodmann’s zone) of children with ASD was revealed [52,53,54]; an increase in miR-320a expression in peripheral blood monocytes was also reported [5,54]. A decrease in the expression level was observed for miR-15b in cerebellar tissue, in peripheral blood samples, in peripheral blood monocytes [5,54], and for miR-195-5p in peripheral blood samples.

Herein, by atomic force microscopy (AFM), we have demonstrated that the size and the number of particles, bio-specifically captured onto the surface of the AFM chips from serum samples of children with ASD, significantly exceed those obtained upon the analysis of samples of healthy volunteers. 

After the incubation in serum samples, the following proteins, involved in the binding of nucleic acids, were revealed on the surface of the AFM chips: guanine nucleotide-binding proteins (GBG10), 28S ribosomal protein S24 (RT24), CC motif chemokine 27 (CCL27, participating in the implementation of chemokine activity), potassium voltage-gated channel subfamily E regulatory beta subunit 5 (KCNE5, participating in the regulation of potassium channels), etc. We did not observe these proteins in the previously performed HPLC-MS/MS analysis of the protein composition of the serum samples of patients with ASD [33].

We assume that nucleic acid binding proteins, which are probably involved in shielding microRNA from serological hydrolases and increasing their stability, can be successfully detected on the AFM chip surface.

Small-sized GBG10 and FAM81B proteins were revealed on the surface of the chips after their incubation in the samples of children with ASD, while these proteins were not revealed on the surface of chips incubated in the samples of apparently healthy volunteers (the control group). GBG10 protein pertains to the family of G proteins, the biological role of which consists in the regulation of transmembrane signaling systems. Unfortunately, the sites of the protein binding to nitrogenous bases have not been characterized in the literature. The structure of GBG10 is not resolved in the PDB database of protein structures (https://www.rcsb.org/, access on 20 July, 2021). However, the authors of the study suggest that the formation of a protein-nucleotide complex between microRNA and GBG10 in the formation of cells (such as glial cells and neurons), and the preservation of the complex in the composition of transport vesicles or LDL particles in the bloodstream is probable.

FAM81B protein is currently insufficiently studied, and its biological activity is yet unclear. Nevertheless, based on the results obtained, we can assume that the surfaces of AFM chips, enriched with target microRNA molecules by bio-specific enrichment (capturing), represent interesting objects of research. On the one hand, the approach proposed herein allows one to visualize and count the formed complexes with a height of 6 to 10 nm, which corresponds in size to macromolecular complexes. The average height of an intact protein makes up about 1.5 nm. On the other hand, mass spectrometry makes it possible to identify protein components in eluates from the surface of AFM chips. It was not possible to identify target microRNA on the chip surface for the following two reasons. Firstly, blood is rich in cations, and microRNAs tend to form multi-cationic modifications with sodium and potassium, which are difficult to be resolved by MALDI-MS. Secondly, the concentration of microRNA on the chip surface is insufficient for MS identification. MicroRNAs are rather difficult to be analyzed by MALDI-MS due to their physicochemical properties: relatively high molecular weight (in comparison with that of tryptic peptides), and difficult ionization.

## 5. Conclusions

The brain cells of children with ASD are characterized by a change in the profile and the total amount of non-coding RNAs, primarily microRNAs. MicroRNAs can cross the blood–brain barrier and circulate in the bloodstream as part of microvesicles, LDL, or macromolecular complexes, thus avoiding enzymatic cleavage by hydrolases and exhibiting longer elimination time. Detection of microRNAs in blood is attractive with respect to the development of novel approaches to minimally invasive serological diagnosis of ASD.

Herein, we have proposed a novel approach, which employs molecular AFM-based fishing, combined with MS analysis. This approach allows one to capture target microRNAs from the volume of analyzed samples onto the surface of AFM chips with immobilized oligonucleotide probes against the target microRNAs, owing to a bio-specific probe/target interaction. In our experiments, AFM chips, bearing an array of sensor areas with surface-immobilized oligonucleotide probes, have been fabricated. The so-captured target microRNAs are subsequently subjected to mass spectrometric analysis of protein components. We have demonstrated that the formation of objects with an average height of 10 nm and 6 nm, occurs after the incubation of the AFM chips in serum samples of ASD patients and conventionally healthy volunteers, respectively. Moreover, the number of particles, visualized on the surface of the chips, incubated in the samples of ASD patients, significantly exceeds that obtained for the samples of conventionally healthy volunteers. As a result of MALDI-TOF MS analysis of protein components of eluates from the surface of the AFM chips, proteins of cellular origin, which are known to be involved in the binding of nucleic acids (guanine nucleotide-binding proteins (GBG10), 28S ribosomal protein S24 (RT24)), and in the implementation of chemokine activity (CC motif chemokine 27 (CCL27), potassium voltage-gated channel subfamily E regulatory beta subunit 5 (KCNE5)) have been revealed. It is interesting to point out that GBG10 and FAM81B proteins were revealed on the surface of the AFM chips after incubation in the samples of ASD patients, while they were not detected after incubation of the chips in the samples of healthy volunteers.

Thus, in our experiments, the formation of macromolecular complexes on the surface of AFM chips has been observed. Our study concerns the revelation of protein components of these complexes. Further development of the combined AFM-MS approach, proposed herein, for the identification of target microRNAs (including the determination of the accuracy of the microRNA detection) is promising. 

## Figures and Tables

**Table 1:** Sequences of specific target seven microRNA probes.

Probes for microRNAs	Secondary Structure	Sequence of Probes
miR-106a-5p	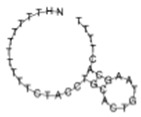	NH_2_-(T)_10_ CT ACC TGC ACT GTA AGC ACT TTT
miR-106b-5p	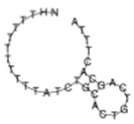	NH_2_-(T)_10_ AT CTG CAC TGT CAG CAC TTT A
miR-19b-3p	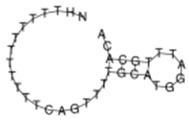	NH_2_-(T)_10_TC AGT TTT GCA TGG ATT TGC ACA
miR-494-5p	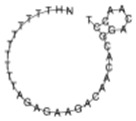	NH_2_-(T)_10_AG AGA AGA CAA CAC GGA CAA CCT
miR-494-3p	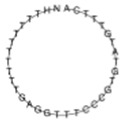	NH_2_-(T)_10_GA GGT TTC CCG TGT ATG TTT CA
miR-15b-5p	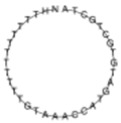	NH_2_-(T)_10_TG TAA ACC ATG ATG TGC TGC TA
miR-320a	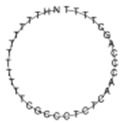	NH_2_-(T)_10_TC GCC CTC TCA ACC CAG CTT TT

**Table 2:** Demographic data of study participants.

Sample No.	Age	Sex	Additional Information
Family 1			
7	8	M	F84.1 Son
6	38	F	Healthy, Mother
8	6	M	Healthy, Son
Family 2			
5	7	M	F84.1 Son
4	34	F	Healthy, Mother
Participants			
11	7	M	F84.1 Son
12	11	M	F84.1 Son
13	11	F	F84.1 Daughter
1	48	F	Healthy, Mother
9	8	M	F84.1 Son
10	11	M	F84.1 Son

**Table 3:** Proteins revealed on the surface of the chips, bearing an array of sensor areas with covalently immobilized oligonucleotide probes, after the incubation in samples of individuals from two comparison groups.

Protein	Frequency of Occurrence in Samples	Function (According to UniProt)	Mw, Da	pI	Aliphatic Index
GBG10	5	Guanine nucleotide-binding proteins (G proteins) are involved into various transmembrane signaling systems as modulators or transducers	7201	7.7	95
FA81B	5	Protein FAM81B	51,990	9.2	82
CDN2C	10	Inhibits cell growth and proliferation	18,116	7.7	95
RT24	5	28S ribosomal protein	19,003	9.5	90
KCNV2	10	Potassium voltage-gated channel subfamily	62,419	6.1	84
CCL27	5	Chemotactic factor, which attracts skin-associated memory T-lymphocytes	12,610	9	100
THA11	10	Transcriptional repressor, playing a central role in embryogenesis and pluripotency of embryonic stem cells	34,433	9.2	71
PSA4	5	Proteasome subunit alpha type-4	29,465	7.6	87
NP1L4	5	Nucleosome assembly protein 1-like 4	42,797	4.6	65
KCNE5	10	Potassium voltage-gated channel subfamily	14,984	5.9	91
RALYL	5	RNA-binding Raly-like protein	32,311	7.7	73

## Data Availability

Proteomic data have been deposited to the Mendeley Data, v1, Isaeva, Arina (2021), “Detection of circulating serum microRNAs and proteins in ASD using functionalized chips for atomic force microscope”, http://dx.doi.org/10.17632/6bpkb4p74j.1.

## Appendix A

**Table A1:** Sequences of 10 probes specific for miRNA associated with ovarian cancer.

Probe No.	Sequence
Probe 1	(NH_2_)-(T)_10_ TC AAC ATC AGT CTG ATA AGC TA
Probe 2	(NH_2_)-(T)_10_ AC AGC CCA TCG ACT GGT GTT G
Probe 3	(NH_2_)-(T)_10_ TC CAA CAC TGT ACT GGA AGA TG
Probe 4	(NH_2_)-(T)_10_ CC ATC TTT ACC AGA CAG TGT TA
Probe 5	(NH_2_)-(T)_10_ TC CAA TGC TGC CCA GTA AGA TG
Probe 6	(NH_2_)-(T)_10_ TC ATC ATT ACC AGG CAG TAT TA
Probe 7	(NH_2_)-(T)_10_ CC AAA CAC TGC TGG GTA AGA CG
Probe 8	(NH_2_)-(T)_10_ TC CAT CAT TAC CCG GCA GTA TTA
Probe 9	(NH_2_)-(T)_10_ CA GAC TCC GGT GGA ATG AAG GA
Probe 10	(NH_2_)-(T)_10_ GA ACT TCA CTC CAC TGA AAT C
